# 7^th^ Brazilian Guideline of Arterial Hypertension: Chapter 2 - Diagnosis and Classification

**DOI:** 10.5935/abc.20160152

**Published:** 2016-09

**Authors:** MVB Malachias, MAM Gomes, F Nobre, A Alessi, AD Feitosa, EB Coelho

## Introduction

The initial assessment of a patient with systemic arterial hypertension (SAH) comprises diagnostic confirmation, suspicion and identification of the secondary cause, and assessment of CV risks. In addition, target-organ damage (TOD) and associated diseases should be investigated. Such assessment comprises BP measurement in the office and/or outside the office, by use of proper technique and validated equipment, medical history (personal and family), physical examination and clinical and laboratory investigation.

General assessments directed to all, and, in some cases, complementary assessments only for specific groups are proposed.

### Measurement of BP

#### In the office

Blood pressure should be measured in all assessments performed by physicians of any specialty and other health care professionals properly trained.

Blood pressure should be measured at least every two years for adults with BP levels ≤ 120/80 mm Hg, and annually for those with BP levels > 120/80 mm Hg and < 140/90 mm Hg.^1^ Manual, semi-automated or automated sphygmomanometers can be used. They should be validated, and calibrated annually following the INMETRO recommendations (Chart 1). The BP should be taken in the arm, with a cuff size adequate to arm circumference (Chart 2). When AH secondary to coarctation of the aorta is suspected, BP should be measured in the lower limbs with proper cuffs.^2^

**Chart 1:** INMETRO ordinances n. 24, of February 22, 1996, for mechanical aneroid sphygmomanometers, and n. 96, of March 20, 2008, for digital electronic sphygmomanometers for non-invasive measurement.

By means of these ordinances, the manufacturers or importers of sphygmomanometers should submit their products to metrological control, defined in Technical Regulation, comprising the following steps:
Technical appreciation of the model – every manufacturer or importer of sphygmomanometers should submit each model manufactured or imported to INMETRO approval, and no change in the sphygmomanometer model approved can be performed without INMETRO’s authorization;
Initial verification – should be performed in all sphygmomanometers inside the manufacturer’s facilities or any other place at INMETRO’s discretion before their release to use;
Periodical verification – should be performed once a year, preferably inside the RBMLQ agency (IPEM) or any other place at INMETRO’s discretion; and
Occasional verification – should be performed at the owner’s request, after device repair and/or maintenance, or when INMETRO deems it necessary.
RBMLQ: Brazilian Legal Metrology and Quality Network; IPEM: State Department of Weights and Measures

**Chart 2:** Correction factors of BP measurement with standard adult cuff ( width, 13 cm, and length, 30 cm), according to patient’s arm circumference

Circumference (cm)	Correction factor (mm Hg)	
	SBP	DBP
26	+5	+3
28	+3	+2
30	0	0
32	-2	-1
34	-4	-3
36	-6	-4
38	-8	-6
40	-10	-7
42	-12	-9
44	-14	-10
46	-16	-11
48	-18	-13

Orthostatic hypotension should be suspected in elderly, diabetic and dysautonomic patients, as well as in those on any antihypertensive medication. Thus, particularly in such conditions, BP should be read with the patient standing for 3 minutes, and orthostatic hypotension being defined as a reduction in SBP > 20 mm Hg or in DBP > 10 mm Hg.^3,4^ Several measurements should be taken, with the patient sitting in a calm and comfortable environment to improve reproducibility and to obtain office BP levels closer to those provided by ambulatory BP monitoring (ABPM) during wakefulness.^5,6^

Procedures recommended for BP measurement:^7^

#### Patient's preparation:

1. Explain the procedure to the patient, who should be left to rest for 3-5 minutes in a calm environment and instructed not to talk during the measurement. Possible doubts should be clarified before or after the procedure.
2. Make sure the patient:
  - does not have a full urinary bladder;
  - did not practice physical exercise in the past 60 minutes;
  - did not consume alcohol, coffee or any food;
  - did not smoke in the past 30 minutes.
3. Position:
  - The patient should be sitting relaxed in a chair, with back supported, legs uncrossed and feet on the floor;
  - The patient's arm should be supported at heart level, not compressed by clothes, with hand palm turned upward.
4. After 3 minutes, BP should be read in the upstanding position in diabetic and elderly patients, or in any other situation at risk for orthostatic hypotension.

#### Steps of BP measurement

1. Determine arm circumference in the middle point between the acromion and olecranon;
2. Select proper cuff size (Chart 3);
  **Chart 3:** Cuff dimensions (bladder width and length) according to arm circumference

Arm circumference (cm)	Cuff denomination	Bladder width (cm)	Bladder length (cm)
≤ 6	Newborn	3	6
6-15	Infant	5	15
16-21	Child	8	21
22-26	Small adult	10	24
27-34	Adult	13	30
35-44	Large adult	16	38
45-52	Thigh	20	42
3. Place the cuff snugly, 2-3 cm above the cubital fossa;
4. Centralize the compressive part of the cuff on the brachial artery;
5. Estimate BP level based on palpation of the radial pulse*;
6. Palpate the brachial artery on the cubital fossa and place the stethoscope's diaphragm without excessive compression*;
7. Inflate cuff rapidly until the estimated SBP level obtained on palpation is exceeded by 20-30 mm Hg*;
8. Proceed to deflation slowly (velocity of 2 mm Hg/second)*;
9. Determine SBP by auscultation of the first sound (Korotkoff phase I), and then, slightly increase the deflation velocity*;
10. Determine DBP when the sounds disappear (Korotkoff phase V)*;
11. Auscultate until 20-30 mm Hg below the last sound to confirm its disappearance, and then proceed to rapid and complete deflation*;
12. If heart beats persist until level zero, determine DBP on the muffling of sounds (Korotkoff phase IV) and write down the values of SBP/DBP/zero*;
13. Take at least two measurements at 1-minute intervals. If the first two are very different, additional readings should be taken. When appropriate, consider the mean value;
14. Measure BP in both arms on the first medical visit and take the higher value as reference;
15. Inform the patient of the BP reading; and
16. Write down the exact BP values, with no rounding, and the arm used for the measurement.
  * Items performed exclusively in the auscultatory technique.

The use of validated and periodically calibrated equipment is paramount.^8^

#### Outside-the-office BP measurement

Outside the office, BP can be measured by use of home BP monitoring (HBPM), following a specific protocol, or by use of 24-hour ABPM.^9,10^

Outside-the-office BP measurements should be stimulated, and can be performed by using a patient's semi-automated device or one belonging to a health care provider. The major advantages of outside-the-office BP measurements are as follows:

- Higher number of BP readings;
- Assessment of the individuals' usual activities;
- Abolition or significant reduction of the 'white-coat effect' (WCE);
- Patients' higher adhesion to diagnosis and follow-up.

The methods usually used to measure BP outside the office are ABPM and HBPM. Both provide similar BP information, but only ABPM assesses BP during sleep. However, both estimate CV risk, and should be considered to assess BP outside the office, provided their indications and limitations are respected.^9,10^ Chart 4 lists the reference values for SAH definition by using office measurements, ABPM and HBPM.^9,10^ Because they are different assessment methods, certain values will be considered for the definition of abnormality. Chart 5 lists the indications for outside-the-office BP measurement by using ABPM and HBPM.

**Chart 4:** Reference values for the definition of AH based on office, ABPM and HBPM measurements

Category	SBP (mm Hg)		DBP (mm Hg)
Office	≥ 140	and/or	≥ 90
ABPM			
Wakefulness	≥ 135	and/or	≥ 85
Sleep	≥ 120	and/or	≥ 70
24 hours	≥ 130	and/or	≥ 80
HBPM	≥ 135	and/or	≥ 85

SBP: systolic blood pressure; DBP: diastolic blood pressure.

**Chart 5:** Clinical indications for outside-the-office BP measurement aimed at diagnosis^9,10,18^

**Clinical indications for ABPM or HBPM**
Suspected WCH
- office stage 1 AH
- office high BP in asymptomatic individuals with no TOD and low total CV risk
Suspected MH
- office BP between 130/85 and 139/89 mm Hg
- office BP < 140/90 mm Hg in asymptomatic individuals with TOD or high total CV risk
Identification of WCE in hypertensive individuals
Wide variation of office BP in the same medical visit or in different visits
Postural, postprandial, siesta or drug-induced hypotension
High office BP or suspected preeclampsia in pregnant women
Confirmation of resistant hypertension
**Specific indications for ABPM**
Significant disagreement between office and outside-the-office BP
Assessment of BP descent during sleep
Suspected AH or usual lack of BP descent during sleep in individuals with sleep apnea, CKD or diabetes
Assessment of BP variability

AH: arterial hypertension; MH: masked hypertension; TOD: target-organ damage; WCE: white coat effect; CKD: chronic kidney disease.

### Measurement of BP in children, elderly, obese and pregnant individuals

#### Children

Measuring BP in children is recommended at all clinical assessments after the age of 3 years, at least once a year, as part of primary pediatric care, and should abide by the standards established for adults.^11^ The interpretation of the BP levels for children and adolescents should consider age, sex and height. The assessment of BP levels according to those variables should be based on specific tables (Chapter 10 of this guideline) or smartphone applications, BP Kids and Ped(z).

#### Elderly

Special aspects of BP measurement in the elderly are due to changes resulting from aging, such as higher frequency of auscultatory gap, which is the absence of sounds during cuff deflation, resulting in falsely low SBP or falsely high DBP readings. The wide BP variability in the elderly throughout 24 hours makes ABPM a useful tool. Pseudohypertension, associated with the atherosclerotic process, can be detected by use of Osler's maneuver, that is, the radial artery remains palpable after cuff inflation at least 30 mm Hg above the reading of radial pulse disappearance.^12^ The higher occurrence of WCE and orthostatic and postprandial hypotension, and the presence of arrhythmias, such as atrial fibrillation, can hinder BP measurement.

#### Obese individuals

The BP measurement of obese patients requires longer and wider cuffs to prevent BP overestimation.^13^ When the arm circumference exceeds 50 cm, and a proper cuff is not available, BP can be taken in the forearm, and the radial pulse should be auscultated.^13^ However, restrictions apply to that practice. Cone-shaped, wide, short arms, where large cuffs do not fit, represent a special difficulty.

#### Pregnant women

The BP should be measured following the same methodology recommended for adults, emphasizing that it can also be taken on the left arm in the left lateral decubitus position at rest, and should not differ from that obtained in the sitting position. Consider Korotkoff's fifth sound as DBP.^14^ White-coat hypertension (WCH) and masked hypertension (MH) are common during pregnancy, and, thus, ABPM and HBPM can be useful for clinical decision making. Chapter 9 provides further information on AH during pregnancy.

### Recommendations for diagnosis and follow-up

**Figure 1:**
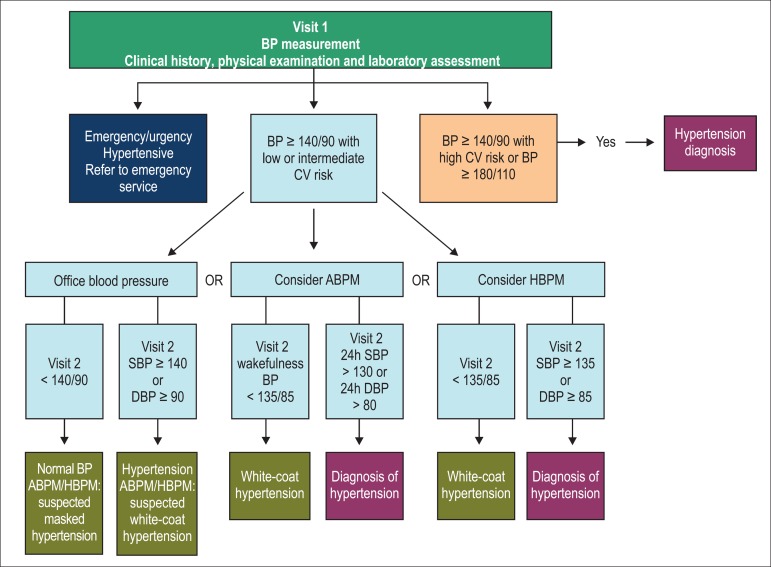
Flowchart for the diagnosis of arterial hypertension (modified from Canadian Hypertension Education Program). Laboratory assessment recommended in Chapter 3. ** Cardiovascular risk stratification recommended in Chapter 3.

To establish the diagnosis and to identify WCH and MH, HBPM and ABPM are recommended (Figure 1).^15^ Another recommendation is suspected AH originating from auto-measurement, when ABPM or HBPM should be used to confirm or rule out the suspected diagnosis of WCH or MH.^16^

### Home BP measurement

Home BP measurement is performed with a specific protocol, and consists in taking three BP readings in the morning, prior to breakfast and medication intake, and three in the evening, before dinner, for five days. Another option is to take two BP measurements in each of those sessions, for seven days.^9,17,18^

Blood pressure levels ≥ 135/85 mm Hg are considered abnormal.

### Ambulatory BP monitoring

Ambulatory BP monitoring allows indirect and intermittent BP recording during 24 hours or longer, while the patient performs their usual chores during wakefulness and sleep. One of its most specific characteristics is the likelihood to identify BP circadian changes, especially during sleep, which has considerable prognostic implications.^19^

Currently, the following BP means are considered abnormal: 24-hour ≥ 130/80 mm Hg, wakefulness ≥ 135/85 mm Hg, and sleep ≥ 120/70 mm Hg.^10,18^

## Classification

The BP limits considered normal are arbitrary. However, the values to classify BP in adults by using casual or office measurements are shown in Chart 6.

**Chart 6:** Classification of BP according to casual or office measurement from 18 years of age onwards

Classification	SBP (mm Hg)	DBP (mm Hg)
Normal	≤ 120	≤ 80
Prehypertension	121-139	81-89
Stage 1 hypertension	140 – 159	90 – 99
Stage 2 hypertension	160 – 179	100 - 109
Stage 3 hypertension	≥ 180	≥ 110
When SBP and DBP are in different categories, the highest should be used to classify BP.		

Isolated systolic hypertension: SBP ≥ 140 mm Hg and DBP < 90 mm Hg, and is should be classified into stages 1, 2 and 3.

### Hypertension

Chart 4 shows the values that define SAH. The BP readings obtained by using different methods have different abnormality levels, therefore, the abnormality levels defined for each method should be considered when establishing the diagnosis of SAH. When using office measurements, the diagnosis should always be validated with repeated readings, under ideal conditions, on at least two occasions, and confirmed by use of outside-the-office measurements (ABPM or HBPM), except for patients with detected TOD.^2,20^ Non-controlled SAH is defined as maintenance of elevated BP, both in and outside the office, by use of either ABPM or HBPM, even under anti-hypertensive treatment.

### Normal blood pressure

**Figure 2:**
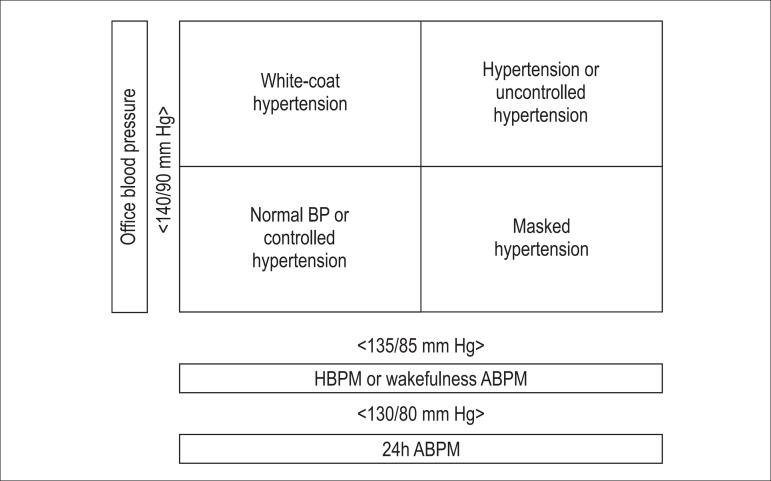
Diagnostic possibilities based on casual BP measurement, ABPM or HBPM. *Consider the diagnosis of prehypertension for casual SBP levels between 121 and 139 and/or DBP between 81 and 89 mm Hg.

Blood pressure is considered normal when office BP levels are ≤ 120/80 mm Hg, and outside-the-office measurements (ABPM or HBPM) confirm those normal readings (Figure 2).^2,21^ Controlled AH is defined as maintenance of controlled BP levels, both in the office and outside it, under anti-hypertensive treatment.

### Prehypertension

Prehypertension is characterized by SBP levels between 121 and 139 and/or DBP levels between 81 and 89 mm Hg. Prehypertensive individuals are more likely to become hypertensive and at higher risk for CV complications than those with normal BP levels, ≤ 120/80 mm Hg, requiring, thus, periodical assessment.^22^

## White-coat effect

The WCE is the BP difference between measurements taken in the office and outside it, if that difference equals at least 20 mm Hg in SBP and/or 10 mm Hg in DBP. It does not change the diagnosis: if normotensive, the individual will remain normotensive; if hypertensive, the individual will remain hypertensive. However, the BP stage can change and/or there might be a false impression of need for change in the therapeutic regimen.

## White-coat hypertension

It is the clinical situation characterized by abnormal office BP levels, but normal BP readings on ABPM or HBPM (Figure 2). Based on four population-based studies, the overall WCH prevalence is 13% (range, 9-16%), and WCH can affect 32% (range, 25-46%) of hypertensive individuals, being more common (55%) in stage 1 hypertensives and affecting 10% of stage 3 hypertensives.^23,24^ However, in terms of prognosis, whether WCH is comparable to normal BP is still controversial, because some studies have shown that its long-term CV risk is intermediate between that of AH and of normotension.^25^

## Masked hypertension

It is characterized by normal office BP, but elevated BP on ABPM or HBPM (Figure 2). The MH prevalence is 13% (range, 10-17%) in population-based studies.^23^ Several factors can elevate outside-the-office BP as compared to office BP, such as young age, male sex, smoking habit, alcohol consumption, physical activity, exercise-induced hypertension, anxiety, stress, obesity, DM, CKD and family history of SAH. The MH prevalence is higher when office BP is borderline.^26^ Meta-analyses of prospective studies report that the incidence of CV events is twice higher in MH than in normal BP, and comparable to that in SAH.^23,26,27^ In diabetic individuals, MH is associated with an increased risk of nephropathy, especially when BP elevation occurs during sleep.^28,29^

Figure 2 shows the different possibilities of BP classification according to its diagnosis, based on the new definition forms.

## Isolated systolic hypertension

Isolated systolic hypertension (ISH) is defined as increased SBP with normal DBP, and, along with pulse pressure (PP), is an important cardiovascular risk factor (CVRF) in middle-aged and elderly patients.^30^

The recommendations are summarized in Chart 7.

**Chart 7:** Summary of the recommendations

Recommendations	Grade of recommendation	Level of evidence
Screening and diagnosis of AH with office BP measurement.	I	B
Diagnosis of SAH based on at least two BP readings per visit, in at least two visits.	I	C
Measuring BP outside the office should be considered to confirm the diagnosis of SAH, identify the type of SAH, detect episodes of hypotension, and maximize the prediction of CV risk.	IIa	B
Outside-the-office BP, ABPM or HBPM can be considered, depending on indication, availability, easiness, cost of use, and, when applicable, patient’s preference.	IIb	C
